# Identification of TMPRSS6 cleavage sites of hemojuvelin

**DOI:** 10.1111/jcmm.12462

**Published:** 2015-02-22

**Authors:** Marco Rausa, Michela Ghitti, Alessia Pagani, Antonella Nai, Alessandro Campanella, Giovanna Musco, Clara Camaschella, Laura Silvestri

**Affiliations:** a Division of Genetics and Cell Biology, IRCCS Ospedale San RaffaeleMilan, Italy; b Vita Salute UniversityMilan, Italy

**Keywords:** iron, HJV, TMPRSS6, serine protease, hepcidin, RGM, homology modelling

## Abstract

Hemojuvelin (HJV), the coreceptor of the BMP-SMAD pathway that up-regulates hepcidin transcription, is a repulsive guidance molecule (RGMc) which undergoes a complex intracellular processing. Following autoproteolysis, it is exported to the cell surface both as a full-length and a heterodimeric protein. *In vitro* membrane HJV (m-HJV) is cleaved by the transmembrane serine protease TMPRSS6 to attenuate signalling and to inhibit hepcidin expression. In this study, we investigated the number and position of HJV cleavage sites by mutagenizing arginine residues (R), potential TMPRSS6 targets, to alanine (A). We analysed translation and membrane expression of HJV R mutants and the pattern of fragments they release in the culture media in the presence of TMPRSS6. Abnormal fragments were observed for mutants at arginine 121, 176, 218, 288 and 326. Considering that all variants, except HJV^R121A^, lack autoproteolytic activity and some (HJV^R176A^ and HJV^R288A^) are expressed at reduced levels on cell surface, we identified the fragments originating from either full-length or heterodimeric proteins and defined the residues 121 and 326 as the TMPRSS6 cleavage sites in both isoforms. Using the N-terminal FLAG-tagged HJV, we showed that residue 121 is critical also in the rearrangement of the N-terminal heterodimeric HJV. Exploiting the recently reported RGMb crystallographic structure, we generated a model of HJV that was used as input structure for all-atoms molecular dynamics simulation in explicit solvent. As assessed by *in silico* studies, we concluded that some arginines in the von Willebrand domain appear TMPRSS6 insensitive, likely because of partial protein structure destabilization.

## Introduction

**Fig 1:**
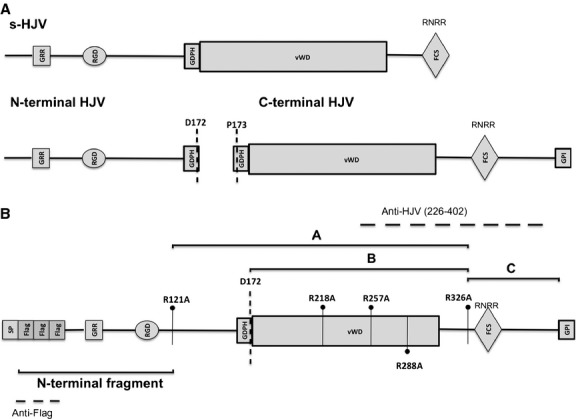
Schematic representation of the hemojuvelin protein. (A) Soluble HJV (s-HJV) 6–8, the C-terminal and the N-terminal portion of the heterodimeric HJV 15 are shown. (B) The Arginine (R) residues mutagenized to Alanine (A) are indicated. Dotted lines indicate the HJV sequence recognized by anti-HJV (raised against a peptide from amino acid 226 to 400) and by anti-FLAG antibodies. The size of fragments A, B and C, released after TMPRSS6 cleavage and recognized by the anti-HJV Ab (see also Fig.3 for comparison), and the HJV N-terminal fragment (from 33 to 121), recognized using the anti-FLAG antibody (see Fig.4 for comparison), are shown as solid line. SP, signal peptide; GRR, glycine-rich domain; RGD (arginine–glycine–aspartic acid), integrin-binding domain; GDPH, autocatalytic cleavage site; vWD, partial von Willebrand domain type D; FCS, furin cleavage site; GPI, glycosylphosphatidylinositol anchor. The autoproteolysis motif is shown by a black dotted line.

Hepcidin is the hepatic peptide hormone which controls iron absorption from the enterocytes and iron release from the stores by degrading the only known cellular iron exporter ferroportin 1. Hemojuvelin (HJV) and TMPRSS6 play a crucial role in regulating hepcidin expression. In hepatocytes, HJV on cell surface (m-HJV) acts as BMP-coreceptor, allowing BMP6 to signal through BMP-SMAD pathway to activate hepcidin transcription 2–5. On the contrary, soluble HJV (s-HJV; Fig.1A) derived from furin cleavage at the RNRR motif 6–8 acts as a decoy molecule, inhibiting hepcidin activation *in vitro* 9,10.

Hemojuvelin belongs to the family of repulsive guidance molecules (RGM) and is mutated in juvenile hemochromatosis 11. The protein is characterized by a signal peptide (SP), a RGD motif, a partial von Willebrand type D (vWD) domain and a glycosilphosphatidylinositol (GPI)-anchor domain 11 (Fig.1A). The protein undergoes partial autoproteolytic cleavage at the GDPH motif 12 to produce a heterodimer that is exposed on the cell surface. The latter is composed by a C-terminal fragment of about 33 kD 13, joined by disulfide bonds 14 to a N-terminal fragment reported to be of about 15–20 kD 12,13,15–17. *In vitro*, HJV is present on the cell surface both as heterodimeric and full-length isoform 14,16. Neogenin, BMPs and TMPRSS6 are HJV interactors. It has been reported that neogenin preferentially binds heterodimeric HJV, while BMP-2 interacts with single-chain HJV species 18. However, we have shown previously that only heterodimeric forms of HJV activates hepcidin *in vitro* 19.

The transmembrane serine protease TMPRSS6 is a major hepcidin inhibitor as its mutations are responsible of Iron Refractory Iron Deficiency Anaemia (IRIDA), characterized by inappropriate hepcidin production 20. Although a formal proof that *in vivo* HJV is the TMPRSS6 substrate is still lacking, *in vitro* the protease cleaves m-HJV into soluble fragments 21, a process that might abolish the signalling and repress hepcidin expression 22,23. TMPRSS6 function is facilitated by the formation of a ternary complex with the transmembrane receptor neogenin on the cell surface 24. The mechanism of TMPRSS6 cleavage and the cleavage sites of HJV are still undefined. One putative cleavage site has been suggested at arginine (R) 288 25. As fragments originating from TMPRSS6 cleavage are multiple 21,26,27, we hypothesize that other sites are cleaved in HJV. To verify this hypothesis, we mutagenized HJV R residues to alanine (A) and analysed the mutant proteins processing, their sensitivity to TMPRSS6 cleavage and their hepcidin activating ability. From the abnormal pattern of fragments released by the HJV variants, here we show that TMPRSS6 cleavage occurs at specific sites and that *in vitro* TMPRSS6 cleaves both the heterodimeric and the full-length m-HJV, originating different C-terminal and the same N-terminal fragments. We identified R residues in the vWD domain whose mutations indirectly cause cleavage-resistance, likely by destabilization of the HJV protein structure. Finally, we better characterized the N-terminal fragments deriving from HJV autoproteolytic process.

## Materials and methods

### Cell culture and expressing vectors

Cell culture media and reagents were from Invitrogen (Carlsbad, CA, USA) and Sigma-Aldrich (St. Louis, MO, USA). HeLa cells were cultured in DMEM and Hep3B in Earle's minimal essential medium, supplemented with 2 mM L-glutamine, 200 U/ml penicillin, 1 mM sodium pyruvate and 10% heat-inactivated foetal bovine serum (FBS) at 37°C in 95% humidifier air and 5% CO_2_.

Hemojuvelin mutants were generated from pcDNA3.1-HJV 19 using the QuickChange II XL mutagenesis (Agilent Technologies, La Jolla, CA, USA), following manufacturer's instructions and using the oligonucleotides listed in Table S1.

TMPRSS6 wild-type and TMPRSS6^MASK^, lacking the serine protease domain, were obtained as described previously 21.

The FLAG-tag HJV expressing vector 2 (HJV^FLAG^), kindly provided by Herbert Lin (Harvard, Boston, MA, USA), was mutagenized as described above.

### Cell surface protein quantification by binding assay

Hemojuvelin surface expression was quantified as described previously 13. In brief, 10^4^ HeLa cells were seeded in 48-well plates and transfected with 0.4 μg of plasmid DNA with 1 μl of Lipofectamine 2000 (Invitrogen). After 12 hrs, the medium was replaced with DMEM supplemented with 2% FBS. After 36 hrs from transfection, cells were fixed with 4% paraformaldehyde. Cells were permeabilized with Triton X-100 in PBS to assess whole HJV expression. Cells were blocked with 5% non-fat milk in PBS, incubated with rabbit anti-HJV (1:1000) and then with the secondary HRP antibody. Peroxidase activity was measured with a HRP substrate (o-phenylenediamine dihydrochloride; Sigma-Aldrich) according to the manufacturer's instructions. The amount of m-HJV was calculated as the ratio between the absorbance of unpermeabilized and permeabilized cells. Background absorbance was subtracted for each sample. Two-tail Student's *t*-test was used for statistical analysis.

### Western blot analysis

HeLa cells were seeded in 100-mm-diameter dishes at 70–80% of confluency and transiently transfected with 13 μg of plasmid DNA and 32.5 μl of liposomal transfection reagent Lipofectamine 2000 (Invitrogen) in 3 ml of Optimem (Invitrogen). After 18 hrs, the medium was replaced with 4 ml of Optimem and 24 hrs later media were collected and concentrated using 5 kD molecular weight (MW) cut-off ultrafiltration (Amicon Ultra; Millipore, Billerica, MA, USA). Cells were lysed, proteins were quantified and total lysates processed for Western Blot analysis as described previously 21 and as detailed in the Data S1.

### Co-immunoprecipitation

HeLa cells were transfected with wild-type or mutant HJV and TMPRSS6^MASK^ (20 μg of total plasmid DNA, ratio 1:1, in a 100-mm-diameter dish). After 36 hrs, cells were lysed in NET/Triton buffer; 500 μg of total lysates were pulled down using the anti-FLAG M2 agarose affinity gel (Sigma-Aldrich) for 2 hrs at 4°C, followed by three washing with NET/Triton buffer. Samples were eluted in Laemmli sample buffer and subjected to SDS-PAGE on 10% acrylamide gel. Immunodetection was performed as described above using rabbit anti-HJV and rabbit anti-FLAG (Sigma-Aldrich) as primary antibodies.

### Cell surface biotinylation assay

HeLa cells, seeded in 100-mm-diameter dishes, were transiently transfected with 13 μg of plasmid DNA as described above. After 18 hrs, the medium was changed with 4 ml of Optimem and 24 hrs later, biotinylation of HeLa surface proteins was performed with EZ-Link NHS-SS-Biotin (Thermo Scientific, Rockford, IL) at 4°C for 30 min., prior to lysis using NET/Triton buffer. Biotinylated proteins were pulled down using the Streptavidin Sepharose High performance beads (GE Healthcare, Buckinghamshire, UK) and equal amount of the resulting samples was loaded on a 10% SDS-PAGE. Immunodetection was performed as described above with anti-HJV and rabbit anti-Pan Cadherin (Abcam, Cambridge Science Park, UK) (for biotin and pull-down normalizations) as primary antibodies.

### Hepcidin promoter-based luciferase assay

A pGL2-basic reporter vector (Promega, Madison, WI, USA), harbouring a 2.9 kb fragment of human hepcidin promoter (Hep-Luc), was used to analyse the hepcidin promoter activity by luciferase assay in Hep3B cells cotransfected with wild-type or mutant HJV expressing vectors, as already reported 19 and as detailed in the Supporting Information section. When indicated, transfected cells were incubated for 3 hrs with 10 ng/ml of recombinant BMP6 (R&D System, Minneapolis, MN). Exogenous HJV levels were measured by qRT-PCR on transfected Hep3B cells (see Data S1). Experiments were performed in triplicate. Two-tail Student's *t*-test was used for statistical analysis.

### Homology modelling and molecular dynamics simulation

I-TASSER server 28 was used to predict the secondary and tertiary structure of HJV. The generated model (G163-L327) was based on the structural coordinates of the RGMb (pdb id=4bq8, chain B and C) that shares a sequence identity of 48% with HJV (RGMc protein). All-atoms Molecular Dynamics simulation in explicit solvent was performed with GROMACS4.6.5 software package 29. Simulation details are in Data S1.

## Results

### Cell surface expression of the HJV variants

We mutagenized 24 (of 29) HJV R residues to alanine (A). Their position in the protein is shown in Figure1B. We did not mutagenize R residues located in the SP, as not present in the mature protein, and in the pro-protein convertase furin cleavage site (FCS), which is not involved in TMPRSS6 cleavage as described previously 21,25.

**Fig 2:**
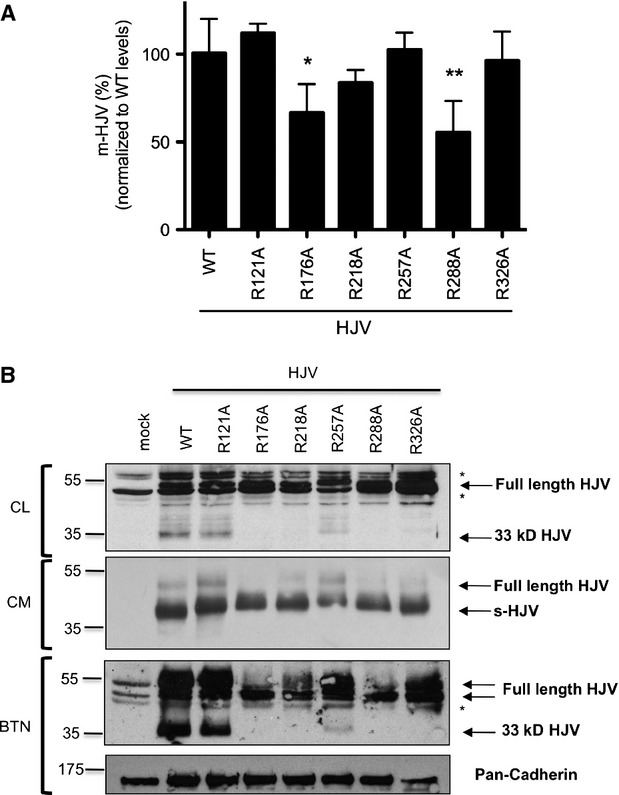
Cell surface expression of HJV variants. (A) HeLa cells, transiently transfected with 0.2 μg of wild-type (wt) or mutant HJV expressing vectors were fixed, permeabilized (to assess whole HJV expression) and incubated with anti-HJV antibody. The amount of membrane-bound HJV was calculated as the ratio between the absorbance of unpermeabilized and permeabilized cells. Background absorbance was subtracted for each sample. HJV^WT^ levels are set to 100% and HJV variants surface expression was normalized to wild-type levels. Statistical significance was calculated on three independent experiments, made in triplicate. Error bars indicate SD. * = *P* < 0.05, ** = *P* < 0.01 (B). HeLa cells were transfected with wild-type (WT) or mutant HJV expressing vectors. Thirty-six hours later, cell culture media were collected and concentrated (CM) and cell surface proteins were biotinylated. Cells were lysed and equal amounts of proteins were pulled down with streptavidin. Whole cell lysate (CL), cell media (CM) and biotinylated proteins (BTN) were loaded on a 10% SDS-PAGE and processed for western blot analysis. The anti-HJV antibody was used to detect HJV and anti-Pan Cadherin to normalize for cell surface expression. Experiments were performed three times. A representative Western blot is shown. The asterisks indicate unspecific bands. Numbers indicate size in kD.

We first assessed whether the HJV variants are correctly translated and reach the cell surface as the wild-type protein. To this aim, we used a previously developed binding assay that quantifies surface HJV in transfected cells 13. Of the 24 available mutants, HJV^R98A^, HJV^R160A^ and HJV^R186A^ are not efficiently translated. All the others reach the cell surface, except HJV^R41A^ (data not shown), HJV^R176A^ and HJV^R288A^ (Fig.2A) that are approximately 35% less expressed on the plasma membrane than wild-type HJV. We then investigated the cellular processing of HJV mutants, including the presence of the 33 kD band, an indicator of the proper autoproteolysis and plasma membrane localization. HJV^R121A^ behaves as the wild-type protein and HJV^R257A^ maintains the ability to undergo autoproteolysis, although at reduced rate (Fig.2B). These variants are thus exported on the cell surface as both heterodimeric and full-length isoforms. HJV^R176A^, HJV^R218A^, HJV^R288A^ and HJV^R326A^ lack the 33 kD band (Fig.2B and Fig. S1), indicating that they do not undergo autoproteolysis and are expressed on the plasma membrane only as full-length isoforms.

All the mutants release s-HJV at the same degree as the wild-type protein, pointing out that the R→A substitution does not affect the furin cleavage process (Fig.2B).

### Identification of TMPRSS6 cleavage sites of HJV

**Fig 3:**
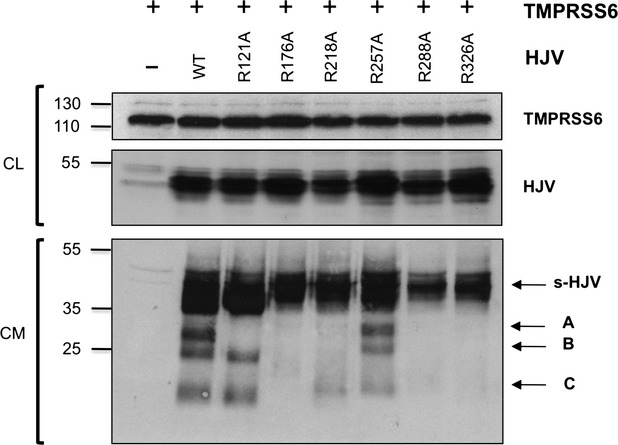
Analysis of TMPRSS6 mediated cleavage on C-terminal HJV. Cell culture media (CM) and total lysate (CL) of HeLa cells cotransfected with TMPRSS6 and WT or HJV variants were loaded onto a 10% SDS-PAGE and processed for western blot analysis. Fragments A, B and C originate from TMPRSS6 cleavage, while s-HJV is not affected by TMPRSS6 (see Fig.1B for fragments interpretation). The anti-HJV antibody was used to detect HJV and anti-FLAG to detect TMPRSS6. Experiments were performed three times. A representative Western blot is shown. Numbers indicate size in kD.

TMPRSS6 releases in the conditioned medium discrete HJV fragments of approximately 30, 25 and 15 kD, indicated as A, B and C respectively (Fig.3) 21, as revealed by the anti-C-terminal HJV antibody 13. These fragments originate from the specific cleavage activity of the serine protease on HJV, as we have repeatedly shown that they are undetectable when a proteolytically inactive variant of TMPRSS6 is co-expressed with HJV 21,26,27. The same pattern of fragments is expected to be modified or absent when R residues, targets of TMPRSS6 cleavage, are mutagenized. A previous study has examined the fragments resulting from TMPRSS6 cleavage using a different antibody 25, which reveals a single evident band of about 36 kD, that could correspond to fragment A in our study. The use of different antibodies and MW markers may explain the size discrepancies in the two studies.

In the presence of TMPRSS6, N-terminal mutants from R41A to R156A (Fig.1), except R121 (see below), behave as the wild-type protein (representative N-terminal mutants are shown in Fig. S2). As the antibody recognizes the C-terminal region of HJV (Fig.1), we conclude that R mutagenesis at the N-terminal part of the protein does not perturb the TMPRSS6-mediated cleavage of the C-terminal portion of HJV.

We observe that HJV^R121A^, when co-expressed with TMPRSS6, releases fragments B and C, but lacks fragment A (Fig.3). In addition, in a less exposed blot (Fig. S3), a band is visible around 40 kD, which could correspond to the abnormal fragment originated from the absence of the cleavage in R121. This clearly indicates that R121 is a TMPRSS6 target site and that its cleavage originates fragment A.

The HJV^R218A^ variant releases only the shortest size (C) fragment, whereas the HJV^R257A^ mutant behaves as the wild-type protein (Fig.3 and Fig. S4), although with lower efficiency, confirming previous finding 25. R substitutions close to the GPI-anchor (positions 329, 344, 345 and 385) and outside the FCS result in normal fragments (data not shown). HJV^R176A^, HJV^R288A^ and HJV^R326A^ that show slightly reduced membrane localization (Fig.2A and B) do not release any fragment in the presence of TMPRSS6 (Fig.3). In theory, each of these positions could be a TMPRSS6 cleavage site. However, based on the fragment size, we favour the arginine at position 326 as cleavage site. Our interpretation takes into account that m-HJV in transfected cells may be either full-length or heterodimeric 13,16. We observed that fragment B is present only in the two mutants (HJV^R121A^ and HJV^R257A^) that undergo autoproteolysis and are exposed as heterodimeric forms (Fig.2B), and that R substitution at position 326 abrogates all fragments (Fig.3). We propose that fragment A originates from the cleavage at position 121 and 326 of the full-length m-HJV and fragment B originates from the cleavage at position 326 of the heterodimeric isoform (from the GDPH motif to amino acid 326; Fig.1B). Fragment C derives from cleavage at position 326 of the C-terminus of both m-HJV isoforms (from amino acid 326 to the GPI-anchor; Fig.1B). Indeed, its intensity is reduced in the HJV variants that do not undergo (HJV^R218A^) or undergo only partial (HJV^R257A^) autoproteolysis (Fig.3) and lack or show reduced heterodimeric m-HJV.

**Fig 4:**
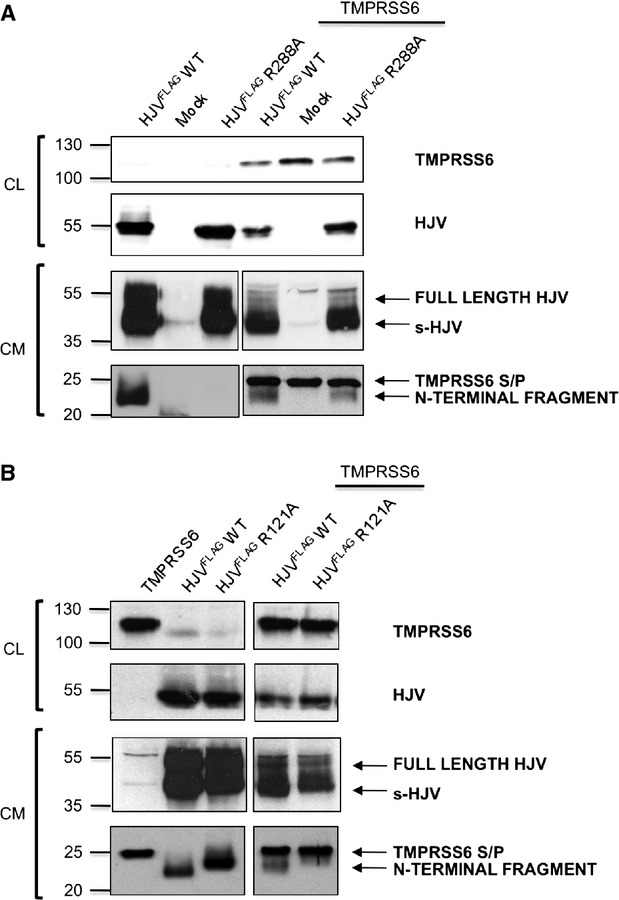
Analysis of TMPRSS6 mediated cleavage on N-terminal HJV. Cell culture media (CM) and lysates (CL) of HeLa cells cotransfected with empty vector (mock), HJV^FLAG WT^, HJV^FLAG R288A^ or HJV^FLAG R121A^ alone or in combination with TMPRSS6 were loaded onto a 10% SDS-PAGE and processed for western blot analysis. The anti-FLAG antibody recognizes both TMPRSS6 (the serine protease domain released in the CM, S/P, and TMPRSS6 in CL) and 3 different HJV isoforms: full-length HJV (CL and CM), s-HJV (CM) and N-terminal HJV fragments derived from autoproteolysis (CM). Experiments were performed three times. A representative Western blot is shown. Numbers indicate size in kD.

To investigate the cleavage products of the N-terminal portion of HJV, in the absence of specific antibodies we took advantage of the HJV^FLAG^ tag construct that in previous functional studies behaves as the wild-type protein 2. HeLa cells transfected with HJV^FLAG WT^ alone release in the culture media the full-length HJV (likely by endogenous PI-PLC cleavage of the GPI-anchor), s-HJV (that originates from furin cleavage; Fig. S5) and a smaller fragment of approximately 22 kD (Fig.4). As the autoproteolytically inactive HJV^FLAG R288A^ variant does not release the latter fragment (Fig.4A), we conclude that the 22 kD band represents the N-terminal region of the heterodimeric surface HJV, originating from the autoproteolytic activity (Fig.4). The size of this fragment is compatible with the reported size of ≈20 kD 14,16,17, the difference being accounted by the three FLAG tags present in our expressing vector.

The same ≈22 kD N-terminal fragment is visible when HJV^FLAG WT^ is coexpressed with TMPRSS6 but is absent in HJV^FLAG R288A^, lacking the autoproteolytic activity (Fig.4A). A band of ≈22 kD appears when the latter mutant is expressed with TMPRSS6 (Fig.4A). This confirms that the fragment originates from full-length HJV and that 121 is the most N-terminal cleavage site. Interestingly, the fragment generated by TMPRSS6 cleavage of full-length HJV and that originating by autoproteolysis share the same MW (from amino acid 33 to 121).

The HJV^FLAG^ ^R121A^ mutant, that undergoes autoproteolysis, releases a larger fragments that migrates slower than those observed in the wild-type protein (about 23–24 kD; Fig.4B). The different size is not because of the protein glycosylation status, as PNGase treatment does not abrogate the difference between wild-type and HJV^FLAG R121A^ fragments (Fig. S6). Likely, the GDPH cleavage is followed by additional cleavage occurring between amino acids 121 and 172 to generate the ≈23–24 kD band.

### All HJV mutants interact with TMPRSS6

To exclude that the altered pattern of fragments observed in some variants was due to impaired interaction with TMPRSS6, we investigated the ability of the studied mutants to interact with the serine protease by co-immunoprecipitation. HeLa cells were cotransfected with HJV^WT^ or R variants in combination with TMPRSS6^MASK^, a serine protease lacking the catalytic domain 22 that was used to prevent HJV degradation 21 and to improve the western blot sensitivity. All the HJV variants examined interact with the TMPRSS6 ectodomain (Fig. S7, Table1), indicating that, in case HJV cleavage fragments were absent, this was not because of lack of interaction with TMPRSS6.

**Table 1:** Summary of the properties of the HJV variants studied in details

Variants	Degradation fragments after TMPRSS6 cleavage	Cell surface expression	Autoproteolytic activity	BMP-coreceptor activity
HJV^WT^	A-B-C	Yes	Yes	Yes
HJV^R121A^	B-C	Yes	Yes	Yes
HJV^R176^	None	35% reduction	No	No
HJV^R218A^	C	Yes	No	Partial
HJV^R257A^	A-B-C	Yes	Yes (reduced)	Yes
HJV^R288A^	None	35% reduction	No	No
HJV^R326A^	None	Yes	No	Partial

### HJV molecular dynamics simulation

**Fig 5:**
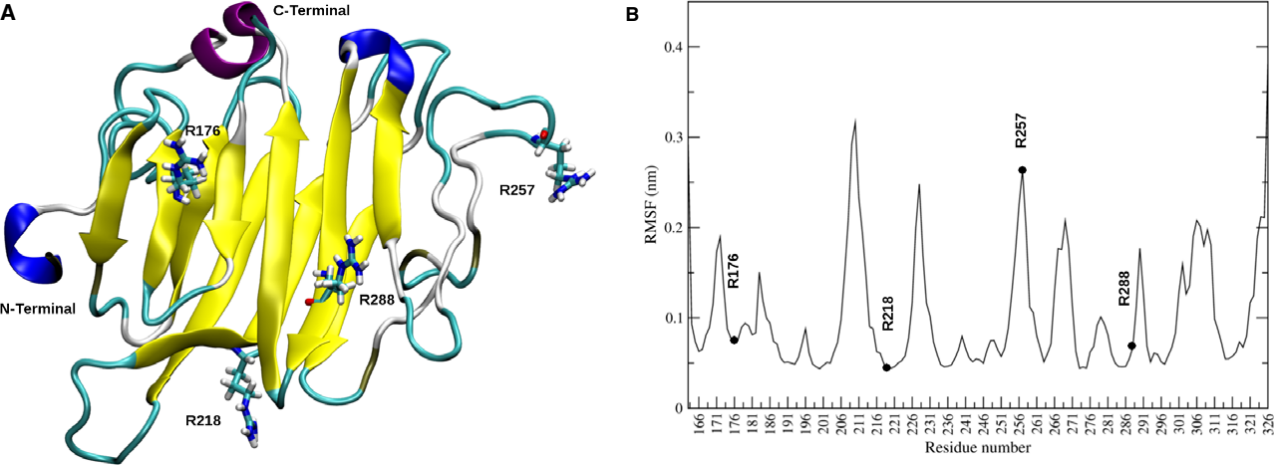
Structural characterization of HJV by means of Molecular Dynamics. (A) Representative HJV snapshot-structure extracted from molecular dynamics simulation shown as cartoon. The extended beta-sheet, the 3–10 helix, the alpha helix, the bridges, the turns and the coils regions are coloured in yellow, blue, purple, tan, cyan and white respectively. The side-chains of R176, R218, R257 and R288 are visualized in sticks. All molecular graphics were produced with VMD 32. (B) The Root Mean Squared Fluctuations (RMSF) of HJV Cα atoms from their time-averaged positions. The RMSF were calculated over the last 80 nsec. of simulation. Cα atoms corresponding to R176, 218, R257 and R288 are visualized as black points.

**Fig 6:**
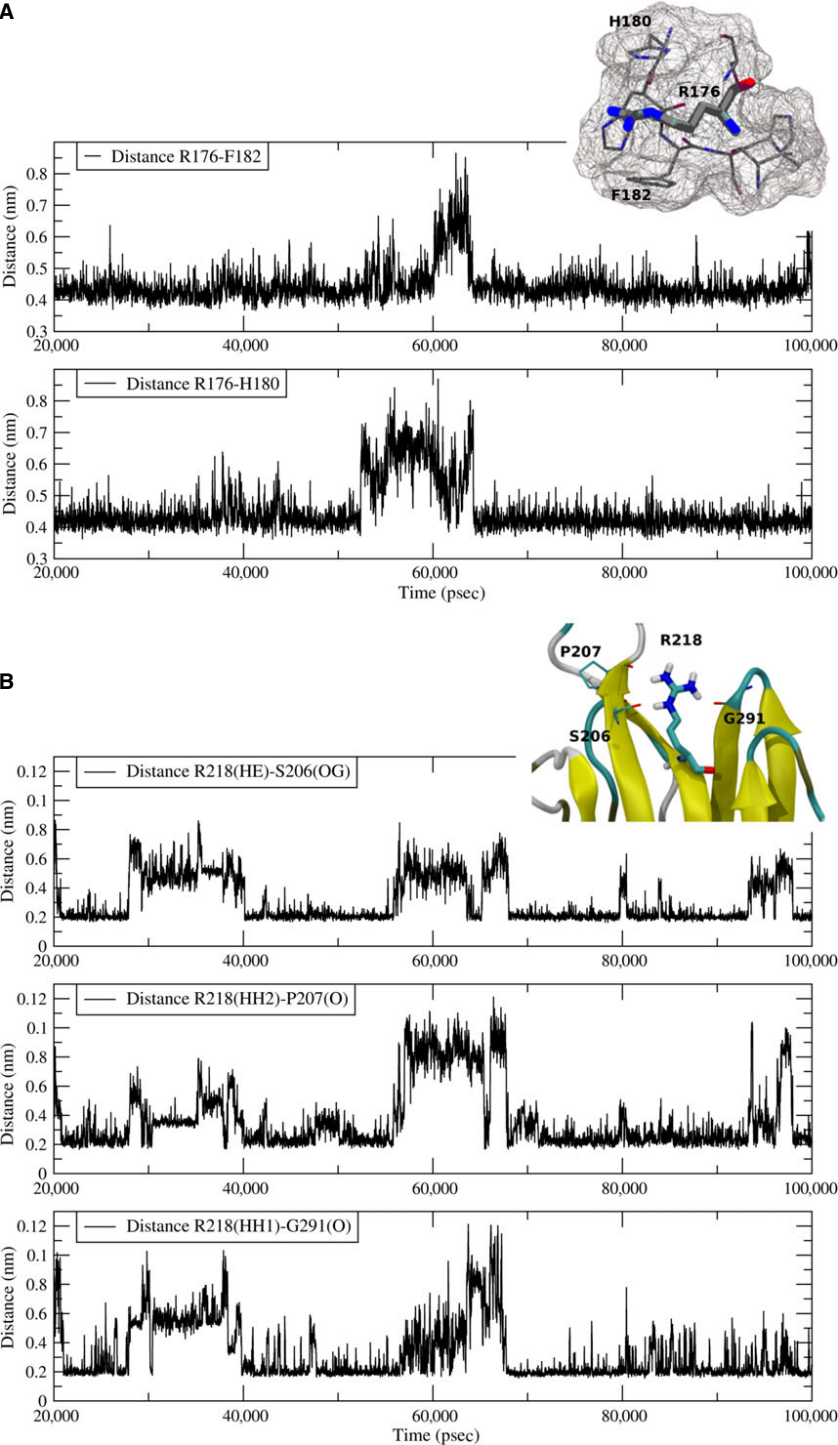
Aromatic cage around R176 and H-bonds network involving R218. (A) Representation of the distance between the centre of mass of R176 and F182 and of R176 and H180 side-chains as a function of time. Distances were monitored during the last 80 nsec. of simulation. In the inset, the surface of the cage-like structure is shown as wireframe. (B) Distances between R218 and S208, P207 and G291 are shown as a function of time. In particular, distance among R218_HE_-S206_OG_, R218_HH2_-P207_O_ and R218_HH1_-G291_O_ was monitored during the last 80 nsec. of simulation.

As the crystallographic structure of RGMb protein (pdb id=4bq8C) 30 shares 48% sequence identity with HJV (RGMc), we used its coordinates to build a homology model of HJV (G163-L327). To investigate the cleavage accessibility of R176, R218, R257 and R288 and to rationalize the functional and structural effects of alanine mutations in these positions, we performed 100 nsec. of molecular dynamics simulation on HJV model and analysed the dynamical behaviour of these arginines during the trajectory. The position of R176, R218, R257 and R288 was mapped on the three-dimensional structure of HJV as shown in Figure5A. In particular, we observed that the backbone atoms of R176, R218 and R288, that are within well defined secondary structure elements (three beta strands) appear quite rigid all along the simulation, as assessed by the low Root Mean Square Fluctuation (RMSF) of their Cα atoms (Fig.5B). Of note, the side-chain of the highly conserved R176 stably interacts with an aromatic cage formed by F182 and H180, creating stabilizing π-cation interactions with the aromatic ring and histidine–arginine pairing respectively (Fig.6A and Fig. S8). Also the side-chain of R218 appears to make relevant stabilizing interactions with neighbouring residues that are located on adjacent strands, as shown by an extended and persistent network of hydrogen bonds with S206, P207 and G291 (Fig.6B). Mutations of both R218 and R176 into alanine are therefore expected to abolish the aforementioned stabilizing interactions, conceivably leading to a local destabilization of the HJV structure. At variance to R218 and R176, R288 does not create any stabilizing interactions, as its side-chain that protrudes out from the beta-sheet is totally exposed to the solvent. Most likely, mutation R288A does not affect the structure stability; however, the high conservation of this residues (Fig. S9) and its involvement in the pathology when mutated into tryptophan 31 strongly suggests a functional role of this residue. Finally, R257 does not have any role in HJV structure, as it is part of a highly fluctuating solvent-exposed loop (Fig.5A), in agreement with its high RMSF value (Fig.5B). Conceivably, a mutation of this residue will not affect stability and might retain the wild-type functionalities.

### Functional characterization of the HJV mutants

To investigate the ability of HJV R variants to act as BMP coreceptors, we analysed basal hepcidin activation on Hep3B cells using a hepcidin promoter luciferase-based assay 19 in the presence of exogenous HJV. Equal transfection efficiency was verified by qRT-PCR (Fig. S10).

**Fig 7:**
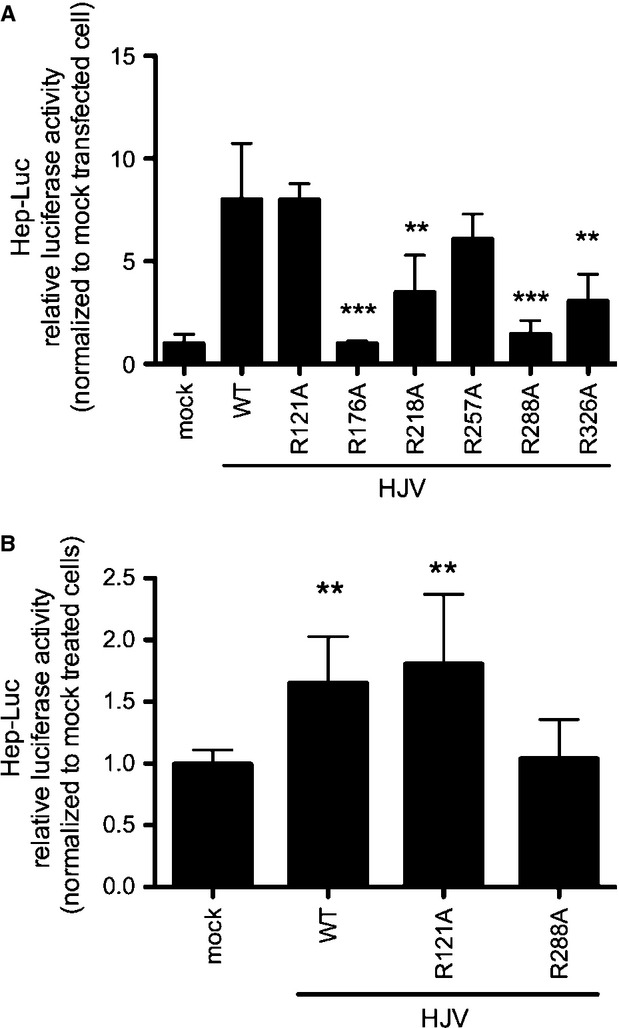
BMP-coreceptor activity of HJV variants. (A) Hep3B cells were cotransfected with a firefly luciferase reporter vector driven by a 2.9 kb proximal hepcidin promoter (Hamp) and pRL-TK (renilla luciferase vector) in combination with empty vector (mock) or wild-type or mutant HJV expressing vectors. Experiments, made in triplicate, were performed three times. (B) Cells were transfected as indicated in (A) and treated with 10 ng/ml BMP6 for 3 hrs. Relative luciferase activity was calculated as the ratio between firefly (reporter) and renilla luciferase activity and expressed as a multiple of activity of cells transfected with the reporter alone. Mean values of mock transfected cells are set to 1 and the values of HJV^WT^ and HJV variants are normalized to mock transfected cells. Experiments, made in triplicate, were performed three times. A representative experiment is shown. Error bars indicate SD. Asterisks indicate the statistical difference between HJV variants and HJV^WT^. **P < 0.01, ***P < 0.001.

HJV^R121A^ and HJV^R257A^ are able to activate hepcidin at the same rate of wild-type HJV, confirming that maintenance of the correct processing and maturation is essential for the HJV-mediated hepcidin activating properties. HJV^R176A^ and HJV^R288A^ do not activate hepcidin, whereas HJV^R218A^ and HJV^R326A^ are only partially active (Fig.7A). To define whether HJV^R121A^ was responsive to BMPs, Hep3B cells, transfected with HJV^WT^, HJV^R121A^ and HJV^R288A^, were treated with BMP6. HJV^R121A^ behaves as HJV^WT^, whereas HJV^R288A^ is insensitive to BMP6 stimulation (Fig.7B and Table1).

## Discussion

Hemojuvelin is a coreceptor in the BMP-SMAD signalling pathway that up-regulates hepcidin in response to increased iron 2, a pathway inhibited by the liver serine protease TMPRSS6. Although the substrate of TMPRSS6 *in vivo* is debated 17, the double knock out mice *Tmprss6*^−/−^ *Hjv*^−/−^ is compatible with *HJV* being downstream *TMPRSS6* 20. *In vitro,* the serine protease cleaves HJV, releasing multiple fragments in the culture media 21,25. To better define the TMPRSS6-mediated HJV processing, we performed an extensive R→A mutagenesis of potential cleavage sites, generating and characterizing 24 different HJV variants. As RGM proteins, HJV is present on the cell surface either as full-length or as a heterodimer protein formed by a short N-terminal peptide (from amino acid 33 to 121; present paper) (Fig.1B) and a large C-terminal peptide (from the GDPH motif to amino acid 400) 15,16. Combining the molecular mass of the fragments generated by TMPRSS6 on wild-type HJV, and the lack of fragments in case of specific HJV R variants, we defined which fragments originate either from full-length and/or from the heterodimer, and identified their corresponding cleavage sites. Among several variants which showed none or only abnormal fragments, HJV^R121A^ and HJV^R326A^ were especially informative. We showed that the largest fragment A is the result of the cleavage at position 121 and 326 of the full-length HJV, fragment B derives from the heterodimeric protein encompassing the region from GDPH cleavage to position 326. The shortest fragment C originates from the C-terminus (from 326 to the GPI-anchor) of both full-length and heterodimer isoforms (Figs1B and 3).

Previous work has indicated position 288 as a potential TMPRSS6 cleavage site 25. Trying to reconcile our data with those reported previously, it is likely that fragment A corresponds to the 36 kD fragment in 25, as the fragment is not released in mutant R288A in both studies. However, we observed that not only 288A but also several substitutions within the vWD domain, except R257A, perturb the fragment pattern. We excluded that this occurs because of lack of interaction of these variants with TMPRSS6. Thus, we investigated the vWD domain by an *in silico* approach to understand whether altering the protein stability could indirectly affect TMPRSS6 cleavage. Taken together, the molecular dynamics simulation and the data regarding the altered HJV processing allowed us to define that the absence or the different pattern of fragments in the medium was likely because of the local protein destabilization rather than to modification of the cleavage target residues. Indeed *in silico* simulations indicate that R176 and R218 mutagenesis would abrogate the stabilizing intramolecular interactions that might influence the cleavage processes. We confirmed that R257 does not have a structural role, as it is located on a flexible solvent-exposed loop and thus its mutation is predicted to be irrelevant to the protein stability. We do not have clear explanation for the apparent TMPRSS6 cleavage-resistance of the 288 residue. However, we observed that R288 is a particularly conserved amino acid (Fig. S9) and that missense change to tryptophan (R288W) causes a severe hemochromatosis disease 31, highlighting the relevance of this residue for HJV function. In addition, we must consider that the R288A mutant is partially defective in membrane targeting. Unfortunately, we could not formulate any reliable prediction on the dynamic behaviour of R121 and R326 as they are outside and at the very end of the modelled structure respectively. Nevertheless, based on fragments size, cleavage at R288 seems unlikely and we favour positions 121 and 326 as TMPRSS6 cleavage sites (Fig.1B).

As the available anti-HJV antibody recognizes only the C-terminal protein, to the aim of confirming our findings, we generated selected variants (R121A and R288A) on a N-FLAG-tagged HJV. This allowed us to confirm that TMPRSS6 cleaves both m-HJV isoforms and to define that there are no N-terminal cleavage sites beyond R121. Moreover, the use of the FLAG-tag construct allowed us to define that the N-terminal fragment derived from autoproteolysis at GDPH undergoes a subsequent more N-terminal rearrangement at R121, likely by other proteases, to generate the correct heterodimeric surface. Accordingly, fragments released by HJV^FLAG R121A^ have higher molecular mass than those released by wild-type HJV.

Interestingly, HJV^R121A^ undergoes autoproteolysis and is correctly expressed on the cell surface. HJV^R121A^ has BMP-coreceptor activity (as HJV^R257A^) in a hepcidin promoter assay both in basal conditions and after BMP6 stimulation. This is of interest, considering that the N-terminal segment of the HJV^R121A^ heterodimeric protein is anomalous and suggests that residues from 121 to 172 are not relevant to the heterodimeric protein function. Pathogenic mutants have been identified at position 168, 170 and 172 31; they do not undergo autoproteolysis and show defective hepcidin activation, thus strengthening the essential role of GDPH autocleavage for HJV functionality.

Autoproteolysis does not occur also in mutants of the vWD domain (HJV^R176A^, HJV^R218A^ and HJV^R288A^). As this is a fundamental process for the correct membrane targeting of the heterodimeric protein 13,19 these variants lose their activity as BMP co-receptors. These results reinforce the idea that R176 and R218 are residues essential for the correct structure stabilization of mature HJV.

In conclusions, we show that TMPRSS6 cleaves HJV at two specific sites both in full-length and heterodimeric protein. Whether the HJV fragments resulting from TMPRSS6 cleavage have a functional role still remains to be demonstrated.

## Supporting Information

**Figure S1**. Cell membrane biotinylation of wild type and HJV arginine variants. **Figure S2**. TMPRSS6 cleavage activity on representative N-terminal HJV arginine variants. **Figure S3**. TMPRSS6 cleavage of HJV^R121A^ variant releases an extra band of approximately 40 kDa. **Figure S4**. Cleavage of HJV^R257A^ by TMPRSS6. **Figure S5**. HJV^FLAG^ releases full length and s-HJV isoforms in the culture media of transfected cells. **Figure S6**. Glycosylation status of autoproteolytically-derived HJV N-terminal fragments. **Figure S7.** All HJV variants interact with TMPRSS6. **Figure S8.** RMSD analysis of HJV backbone atoms. **Figure S9.** HJV sequence analysis. **Figure S10.** *HJV* expression levels in transfected Hep3B cells. **Table S1.** Oligonucleotides used to generate the HJV variants analyzed in details in this study. **Data S1**. Detailed materials and methods.
