# Supplementary material for: Identification of TMPRSS6 cleavage sites of hemojuvelin
Source: J Cell Mol Med. 2015 Feb 22;19(4):879–88. doi: 10.1111/jcmm.12462 (PMC4395201; doi:10.1111/jcmm.12462)

**SUPPLEMENTARY INFORMATION**

**MATERIALS AND METHODS**

**Western blot analysis**

Transfected cells were lysed in NET/Triton buffer (150mM NaCl, 5mM EDTA, and 10mM Tris [pH 7.4] with 1% Triton X-100) and proteins were quantified by using the Bio-Rad Protein Assay (Bio-Rad, Hercules, CA); equal amount of total proteins (50 μg) were loaded on to 10% SDS-PAGE and transferred to Hybond C membrane (Amersham Bioscences Europe GmbH, Freiburg, Germany) by standard western blotting technique. Membranes were blocked with 2% ECL Advance Blocking Agent (Amersham Bioscences) or ECL Prime Blocking Agent (Amersham Bioscences) in TBS (0.5M Tris-Hcl [pH 7.4] and 0.15M NaCl) containing 0.1% Tween-20 (TBST), incubated 2 hrs with rabbit anti-HJV (1:1000) (Silvestri, Blood 2008) and rabbit anti-FLAG (1:1000) (Santa Cruz CA). After washing with TBST, blots were incubated 1 hr with goat HRP-conjugated secondary antirabbit and developed with a chemiluminescence detection kit (ECL, Amersham Bioscences). For deglycosylation, 100 μg of proteins from cell medium were incubated with PNGase F (New England Biolabs) according to manifacturer’s instruction. Proteins were precipitated using cold acetone and then resuspended using Lammli sample buffer. Proteins were then loaded on a 12% SDS-Page. Immunodetection was performed as described above with anti-Flag.

**Hepcidin promoter based luciferase assay**

Hep3B cells, seeded at 70%-80% of confluency, were transiently transfected with the hepcidin promoter luciferase reporter construct (0.25 μg) combined with pRL-TK Renilla luciferase vector (Promega), to control for transfection efficiency, and with expressing vectors encoding wild type or mutant HJV (0.05 μg). Eighteen hours after transfection the medium was replaced with EMEM supplemented with 2% FBS. After 24 hours of serum starvation cells were lysed. When indicated, cells were treated with 10ng/ml of recombinant BMP6 (R&D system) for three hours and then processed. Luciferase activity was determined according to manifacture’s instructions (Dual Luciferase Reporter Assay, Promega). Relative luciferase activity was calculated as the ratio between firefly (reporter) and Renilla luciferase activity, and expressed as multiple of the activity of cells transfected with the report alone. Experiments were performed in triplicate. The student’s t test was used for statistical analysis.

**Quantitative reverse transcriptase polymerase chain reaction**

Total RNA was extracted from Hep3B cells transfected as described above in “**Hepcidin promoter based luciferase assay”.** One μg of RNA, extracted with RNeasy mini kit (Quiagen), was used for first-strand synthesis of cDNA with the High Capacity cDNA Reverse Transcription kit (Applied Biosystems, Warrington UK), according to the manufacturer’s instructions. Gene expression levels were measured by quantitative real-time PCR using Sybr Green Master Mix (Applied Biosystem). Primers used for qRT-PCR are: *HJV* sense: 5’-tgacttcctctttgtccaagc-3’; *HJV antisense*: 5’ tgcatgttcttaaatatgatggtga-3’; *HPRT1* sense: 5’-GACCAGTCAACAGGGGACAT-3’; *HPRT1* antisense: 5’-GTGTCAATTATATCTTCCACAATCAAG-3’.

**Homology Modelling and Molecular Dynamics simulation**

Protonation states were chosen to correspond to neutral pH. The system was solvated terminally capped in a 6x7x2 nm^3^ box containing 9.194 TIP3P [[1](#_ENREF_1)] water molecules. The net charge of the proteins was neutralized with sodium ions. The Amber ff99SB-ILDN [[2](#_ENREF_2)] force field was used to represent all systems. The system was initially subjected to energy minimization, followed by equilibration at 300K with 500 ps, during which water molecules and protein heavy atoms were position-restrained. In the second phase (1ns), both water molecules and protein atoms were unrestrained to allow system relaxation. All bonds involving hydrogen atoms were constrained with the SHAKE algorithm [[3](#_ENREF_3)]. Equilibration was performed using the Nosé-Hoover thermostat [[4](#_ENREF_4)] and a Berendsen barostat [[5](#_ENREF_5)] to maintain a constant temperature (300K) and a constant pressure (1 atm). The unrestrained production runs were then simulated for 100 ns in a NPT ensemble, using the V-Rescale thermostat [[6](#_ENREF_6)] and a Parrinello-Rahman barostat [[7](#_ENREF_7)]with a relaxation time of 2 ps. A cutoff of 9 Å was used for the Lennard-Jones interaction and the short-range electrostatic interactions. Verlet cutoff scheme was introduced [[8](#_ENREF_8)]. The smooth particle mesh Ewald method [[9](#_ENREF_9)] with a fourth-order interpolation scheme was used to compute the long-range electrostatic interactions. The pairlists were updated every 10 fs with a cutoff of 9 Å. A uniform integration step of 2 fs was used. Simulation was performed using 156 cores, adopting a Dynamic load balancing option. Trajectory obtained from the NPT run was used for subsequent data analysis. Simulations analysis was performed using the GROMACS package tools. Simulation stability was ascertained monitoring the Root Means Square Deviation (RMSD) along the simulation (**Figure S8**).

**Supplemental References**

1. **Jorgensen WL, Chandrasekhar J, Madura JD, *et al***. Comparison of Simple Potential Functions for Simulating Liquid Water. *J Chem Phys*. 1983; 79: 926.

2. **Lindorff-Larsen K, Piana S, Palmo K, *et al***. Improved side-chain torsion potentials for the Amber ff99SB protein force field. *Proteins*. 2010; 78: 1950-8.

3. **Ryckaert JP, Ciccotti G, Berendsen HJC**. Numerical integration of the cartesian equations of motion of a system with constraints: molecular dynamics of n-alkanes. *J Comput Phys*. 1977; 23: 327-41.

4. **Cheng A, Merz KMJ**. Application of the Nosé-Hoover Chain Algorithm to the Study of Protein Dynamics. *J Chem Phys*. 1996; 100: 1927-37.

5. **Berendsen HJC, Postma JPM, van Gunsteren WF, *et al***. Molecular-Dynamics with Coupling to an External Bath. *J Chem Phys* 1984; 81: 3684–90.

6. **Bussi G, Donadio D, Parrinello M.** Canonical sampling through velocity rescaling. *J Chem Phys*. 2007; 126: 014101.

7. **Parrinello M, Rahman A.** Polymorphic transitions in single crystals: A new molecular dynamics method. *J Appl Phys*. 1981; 52: 7182-90.

8. **Páll S, Hess B.** A flexible algorithm for calculating pair interactions on SIMD architectures. *Comput Phys Commun*. 2013; 184: 2641-50.

9. **Darden T, York D, Pedersen L.** Particle Mesh Ewald-an N.Log(N) method for Ewald sums in large systems. *J Chem Phys*. 1993; 98: 10089-92.

10. **Berezin C, Glaser F, Rosenberg J, *et al.*** ConSeq: the identification of functionally and structurally important residues in protein sequences. *Bioinformatics*. 2004; 20: 1322-4.

**Table S1. Oligonucleotides used to generate the HJV variants analyzed in details in this study**

| Amino acid substitution | Sense | Antisense |
| --- | --- | --- |
| R121A | CCAGCACAACTGCTCCGCTCAGGGCCCTACAGC | GCTGTAGGGCCCTGAGCGGAGCAGTTGTGCTGG |
| R176A | GGACCCCCATGTGGCTAGCTTCCACCATCACTTTC | GAAAGTGATGGTGGAAGCTAGCCACATGGGGGTCC |
| R218A | GGCCAACGCTACCGCCACCGCTAAGCTCACCATC | GATGGTGAGCTTAGCGGTGGCGGTAGCGTTGGCC |
| R257A | CTATCAATGGAGGTGACGCTCCTGGGGGATCCAG | CTGGATCCCCCAGGAGCGTCACCTCCATTGATAG |
| R288A | GCACAACTATAATCATTGCTCAGACAGCTGGGCAG | CTGCCCAGCTGTCTGAGCAATGATTATAGTTGTGC |
| R326A | GCCCTCCAAGTCAGGCTCTCTCTCGATCAGAG | CTCTGATCGAGAGAGAGCCTGACTTGGAGGGC |

**LEGEND TO SUPPLEMENTAL FIGURES**

**Figure S1. Cell membrane biotinylation of wild type and HJV arginine variants**

Short exposure of blot of **Figure 2B** (BTN panel). Arrows indicate the full length and the 33 kDa HJV. Numbers indicate size in kDa.

**Figure S2. TMPRSS6 cleavage activity on representative N-terminal HJV arginine variants**

Cell culture media (CM) of HeLa cells, cotransfected with TMPRSS6 and WT or HJV^R95A^, HJV^R153A^ and HJV^R156A^ expressing vectors were loaded onto a 10% SDS-PAGE and processed for western blot analysis. Fragments A, B and C are derived from TMPRSS6-mediate m-HJV cleavage, while s-HJV is not affected (see **Figure 1** for fragments interpretation). Numbers indicate size in kDa.

**Figure S3. TMPRSS6 cleavage of HJV^R121A^ variant releases an extra band of approximately 40 kDa**

Short exposure of blot of **Figure 3** (CM). The band around 40 kDa in HJV^R121A^ lane probably originates from 33-326 amino acids. Asterisks indicate unspecific band. Numbers indicate size in kDa.

**Figure S4. Cleavage of HJV^R257A^ by TMPRSS6**

HeLa cells were transfected as indicated in **Figure 3**. Fragments A, B and C that originate from TMPRSS6 cleavage of HJV are indicated. Fragments originating from HJV^R257A^ migrate as those from HJV^WT^. A representative Western blot is shown. Numbers indicate size in kDa.

**Figure S5. HJV^FLAG^ releases full length and s-HJV isoforms in the culture media of transfected cells.**

Concentrated media of cells transfected with empty vector (mock) or HJV^FLAG^ ^WT^, HJV^FLAG^ ^R121^ and HJV^FLAG^ ^R288A^ were loaded onto a 10% SDS-PAGE, processed for western blot analysis and incubated with anti-FLAG antibody. In the blot the two bands of full length HJV and s-HJV are distinguishable. Numbers indicate size in kDa.

**Figure S6. Glycosylation status of autoproteolytically-derived HJV N-terminal fragments**

Hela cells were transfected with empty vector (mock), HJV^FLAG^ ^WT^ or HJV^FLAG R121A^. Cell culture media (CM) were collected and concentrated. Equal amount of protein was deglycosylated with PNGase F (+). Control samples were not deglycosylated (-). Proteins were precipitated using cold acetone, resuspended in Laemmli sample buffer and then loaded onto a 12% SDS-PAGE, blotted and incubated with anti-FLAG antibody. The detected band corresponds to the autoproteolytically-derived N-terminal fragment of HJV. Numbers indicate size in kDa.

**Figure S7. All HJV variants interact with TMPRSS6**

HeLa cells were cotransfected with TMPRSS6^MASK^ and HJV^WT^ or mutant HJV. Equal amount of total lysate was pulled down with anti-FLAG resin and then loaded onto a 10% SDS polyacrylamide gel electrophoresis, blotted and incubated with the anti-HJV antibody. Cell lysates (CL) were loaded onto a 10% SDS-PAGE, blotted and incubated with anti-HJV and anti-FLAG antibody. Arrows indicate full length HJV. Experiments were performed three times. A representative Western blot is shown. Numbers indicate size in kDa.

**Figure S8. RMSD analysis of HJV backbone atoms.**

Root Mean Square Deviation (RMSD) of HJV as function of time showing simulation stability. The RMSD was calculated on the backbone atoms after fitting on the backbone atoms and using the starting structure as reference.

**Figure S9. HJV sequence analysis.** The Consurf server [[10](#_ENREF_10)] was used to identify functionally and structurally important HJV residues. Multiple alignment was built using MAFFT and the homologues were collected from UNIREF90. The homologues were searched exploiting CS-BLAST with a maximal and minimal ID between sequence of 95 % and 35 % respectively. Calculation was performed on 119 unique sequences. R176, R218, R257 and R228 are highlighted as black dots.

**Figure S10. *HJV* expression levels in transfected Hep3B cells**

Hep3B cells were transfected with empty vector or WT and HJV arginine variants (as described in **Figure 7A**) and RNA was isolated 36 hours post transfection. *HJV* mRNA expression was evaluated by qRT-PCR. mRNA expression ratio was normalized relative to housekeeping *HPRT1*. Error bars indicate SD. Experiments, made in triplicate, were performed three times.


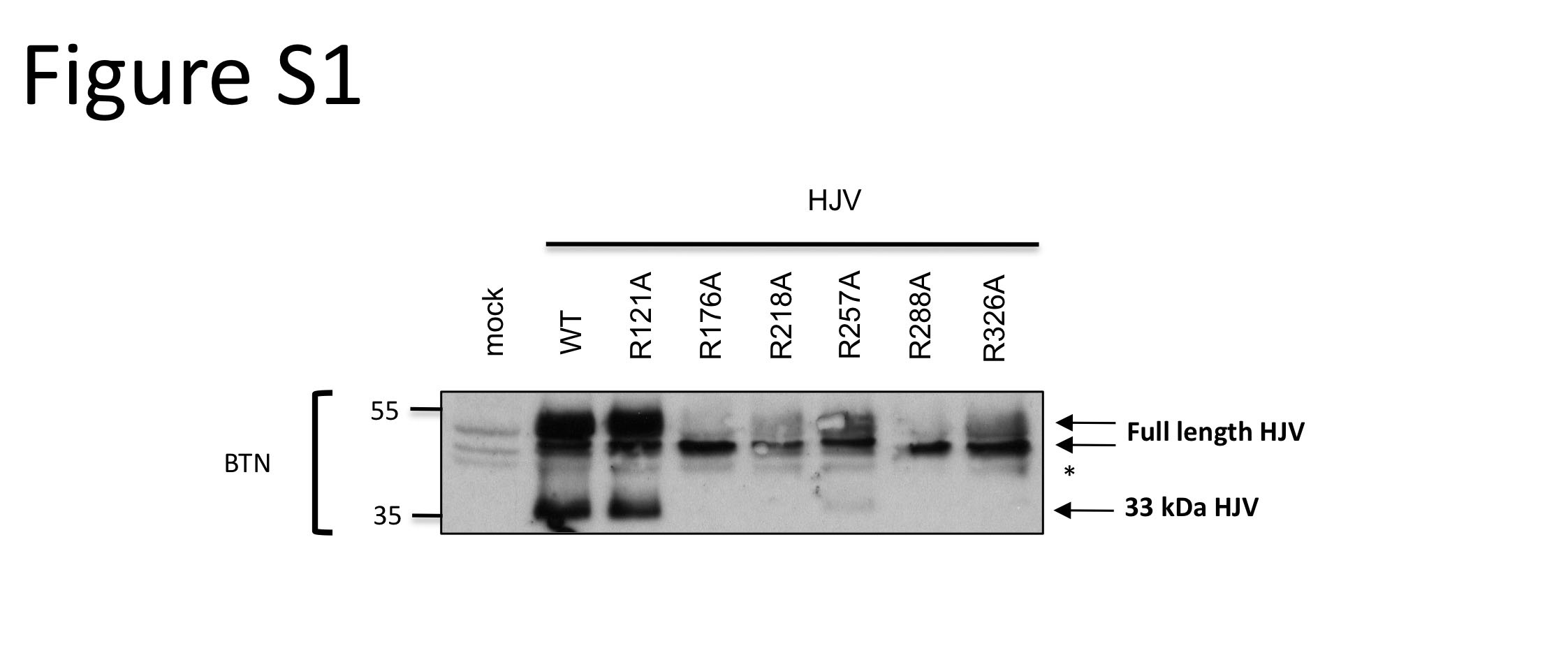


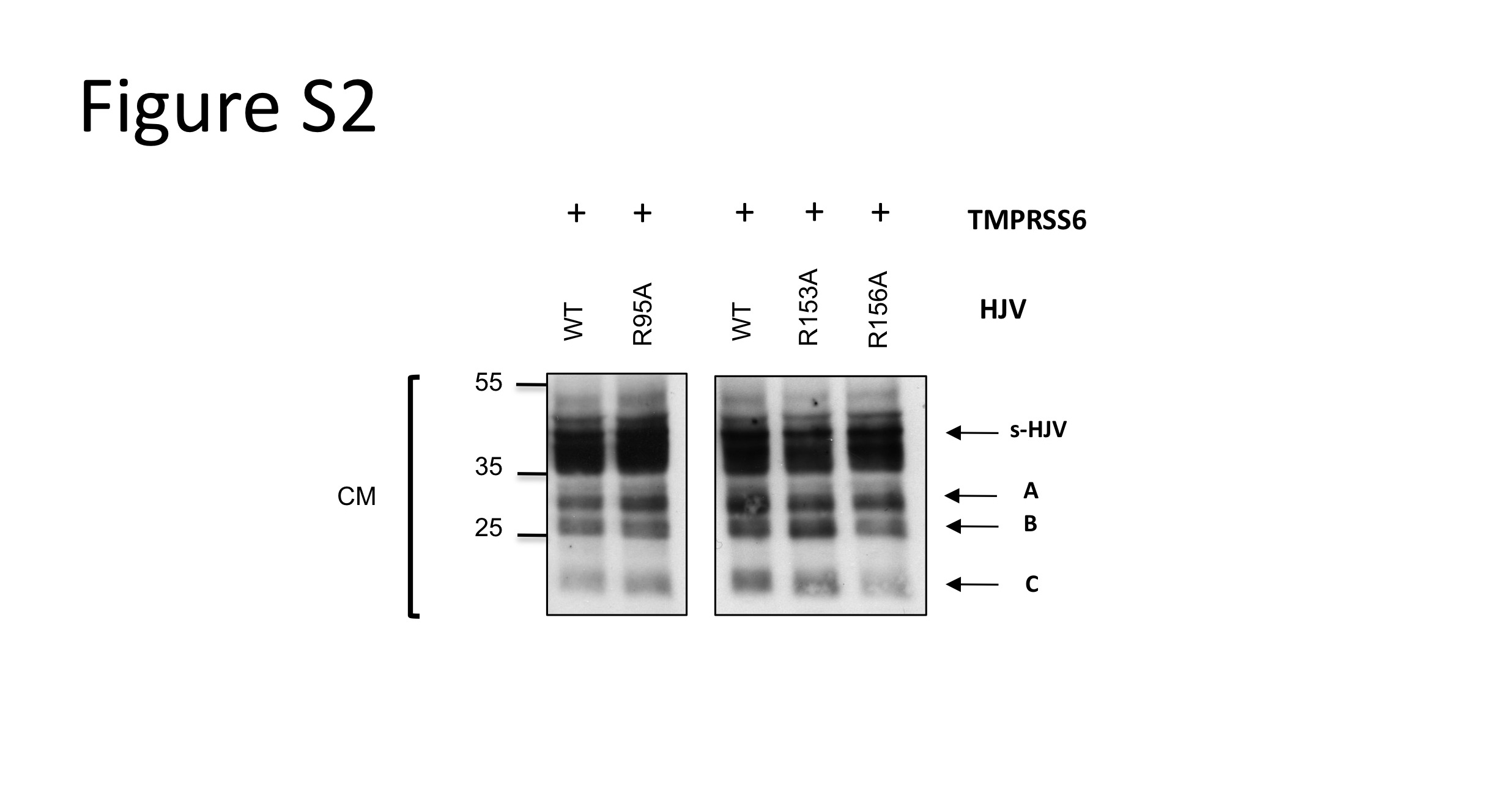


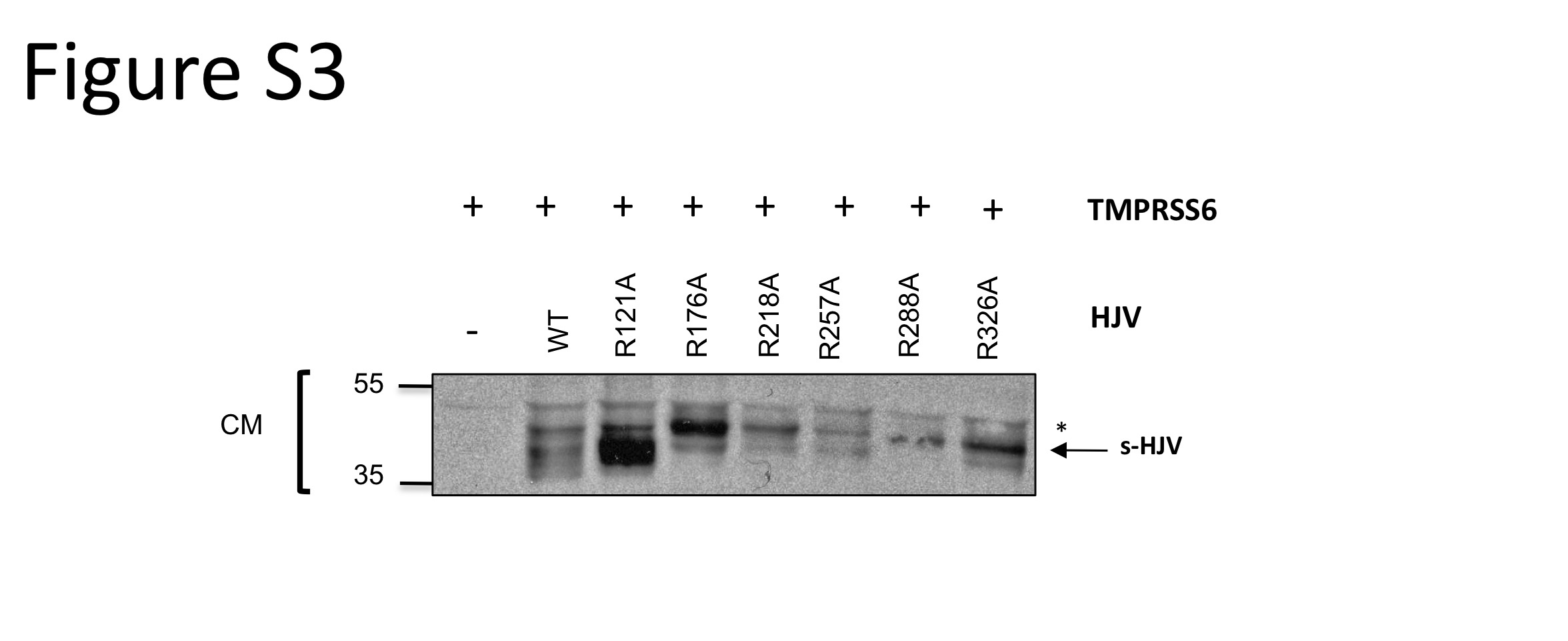


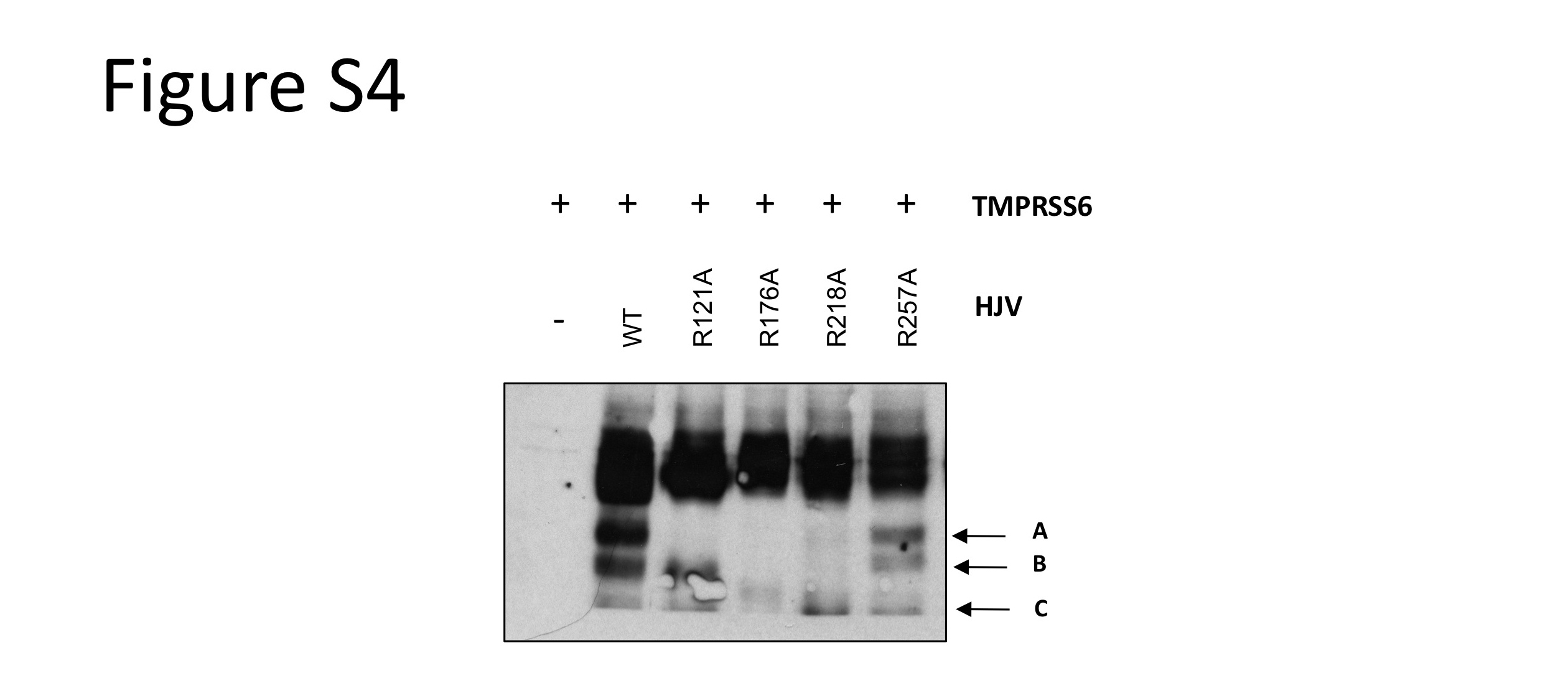


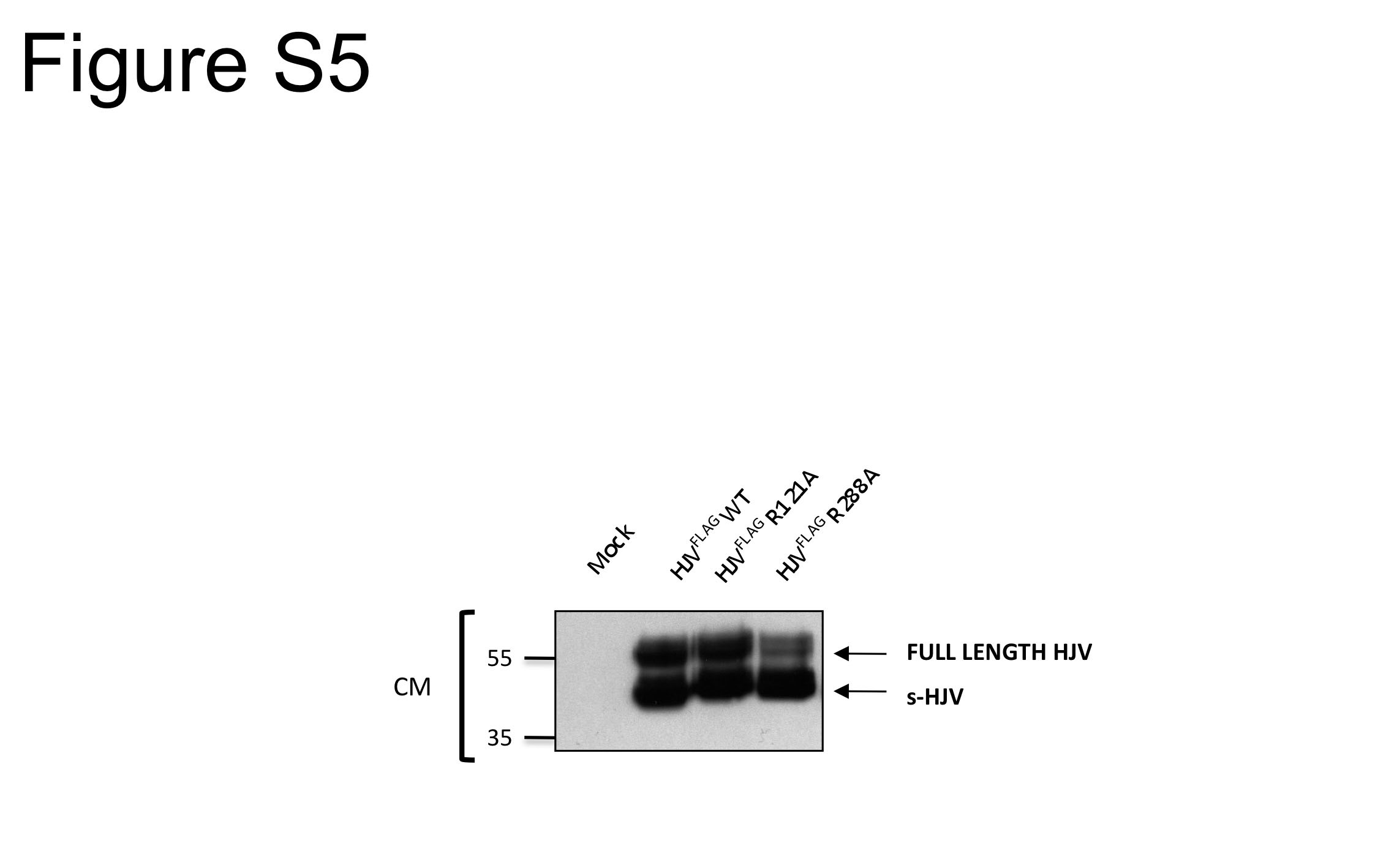


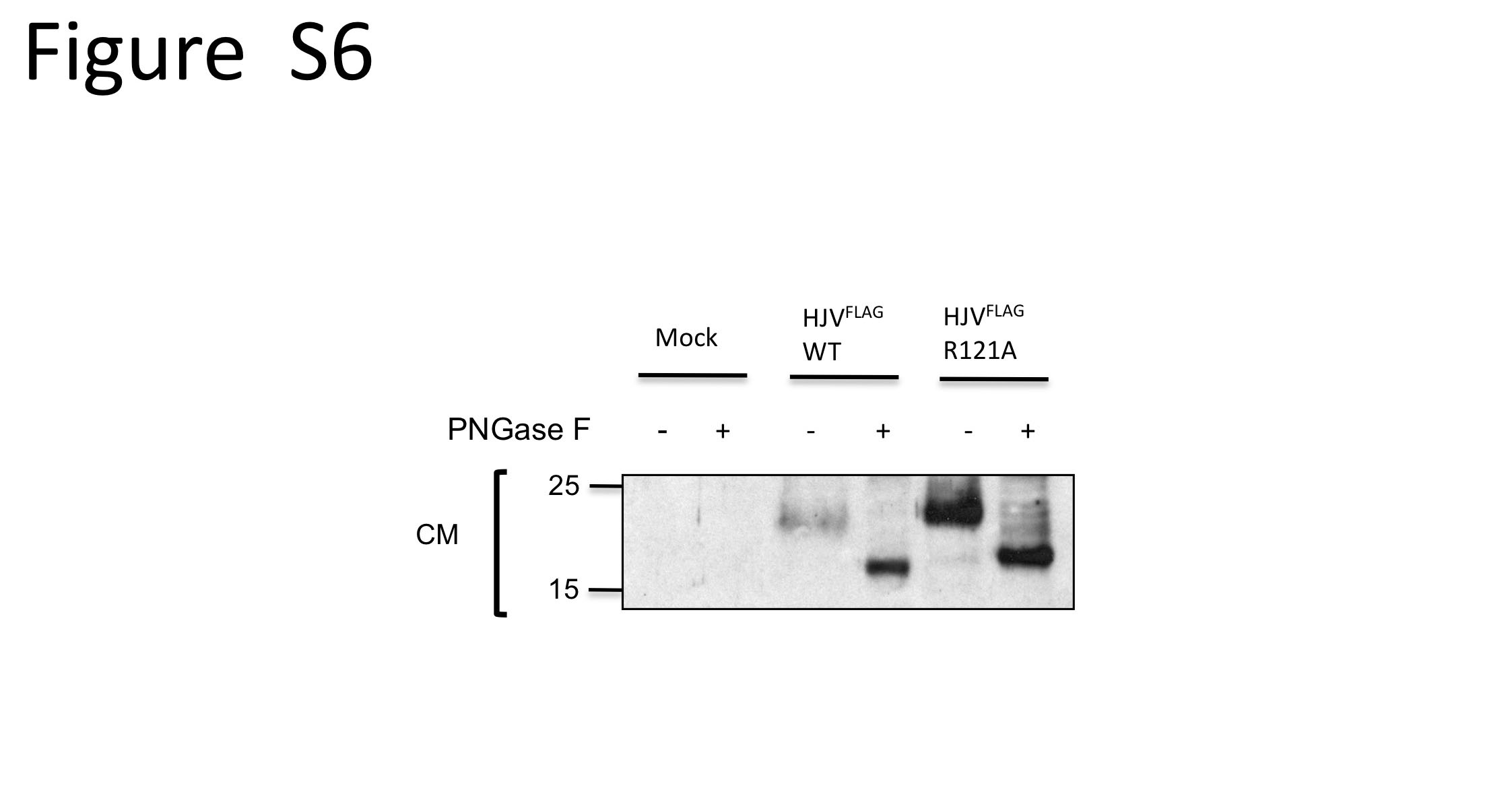


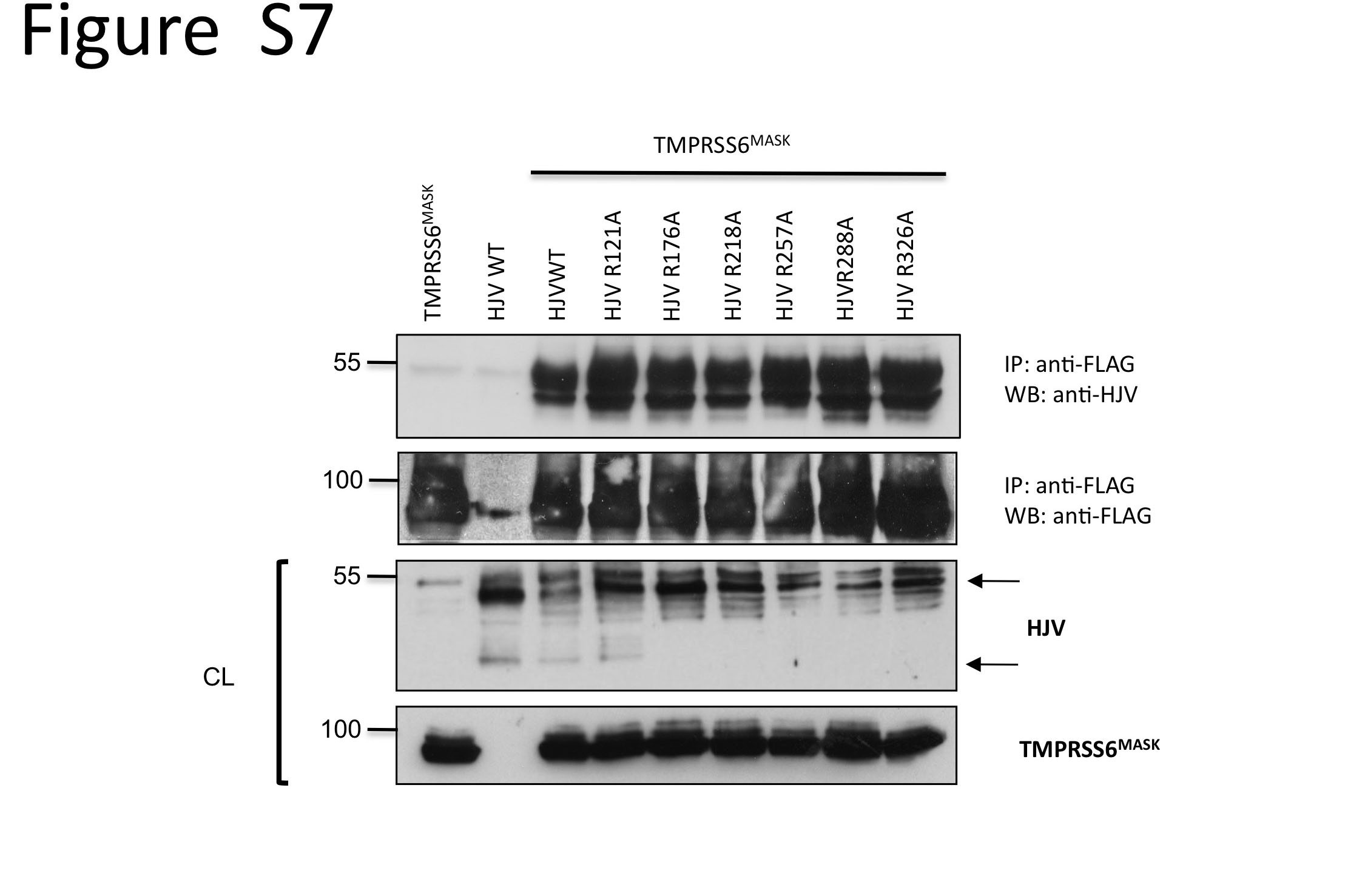


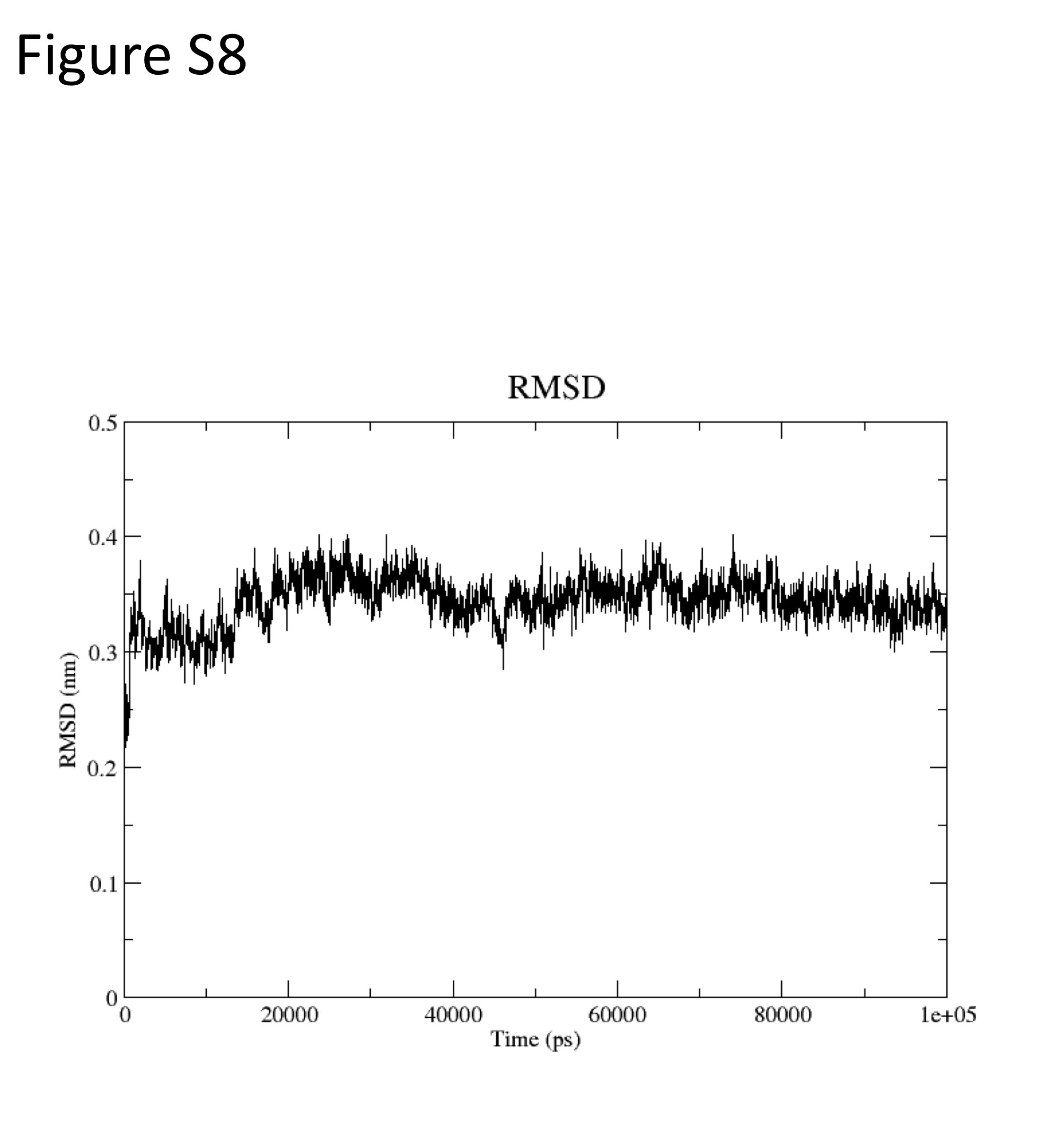


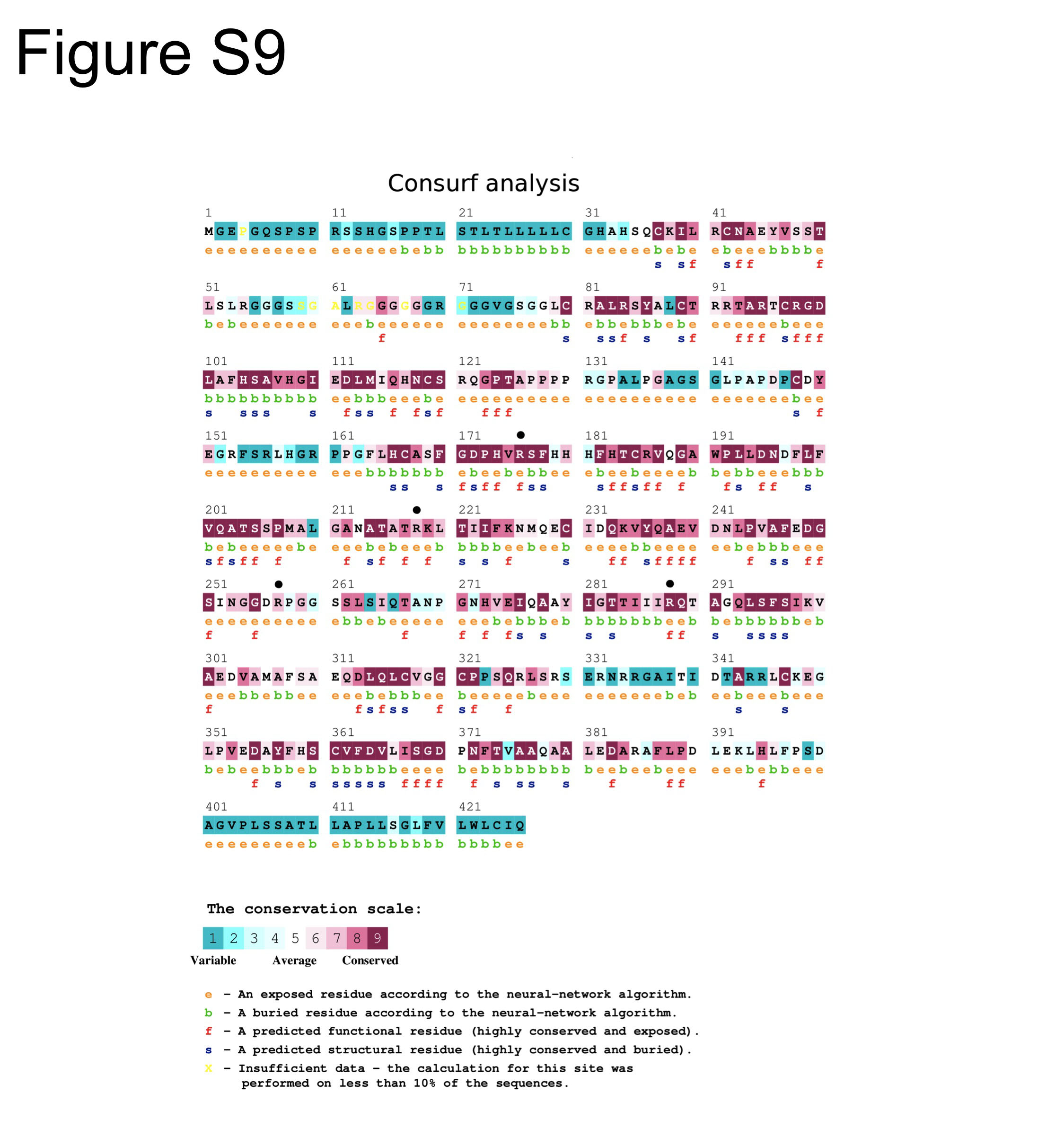


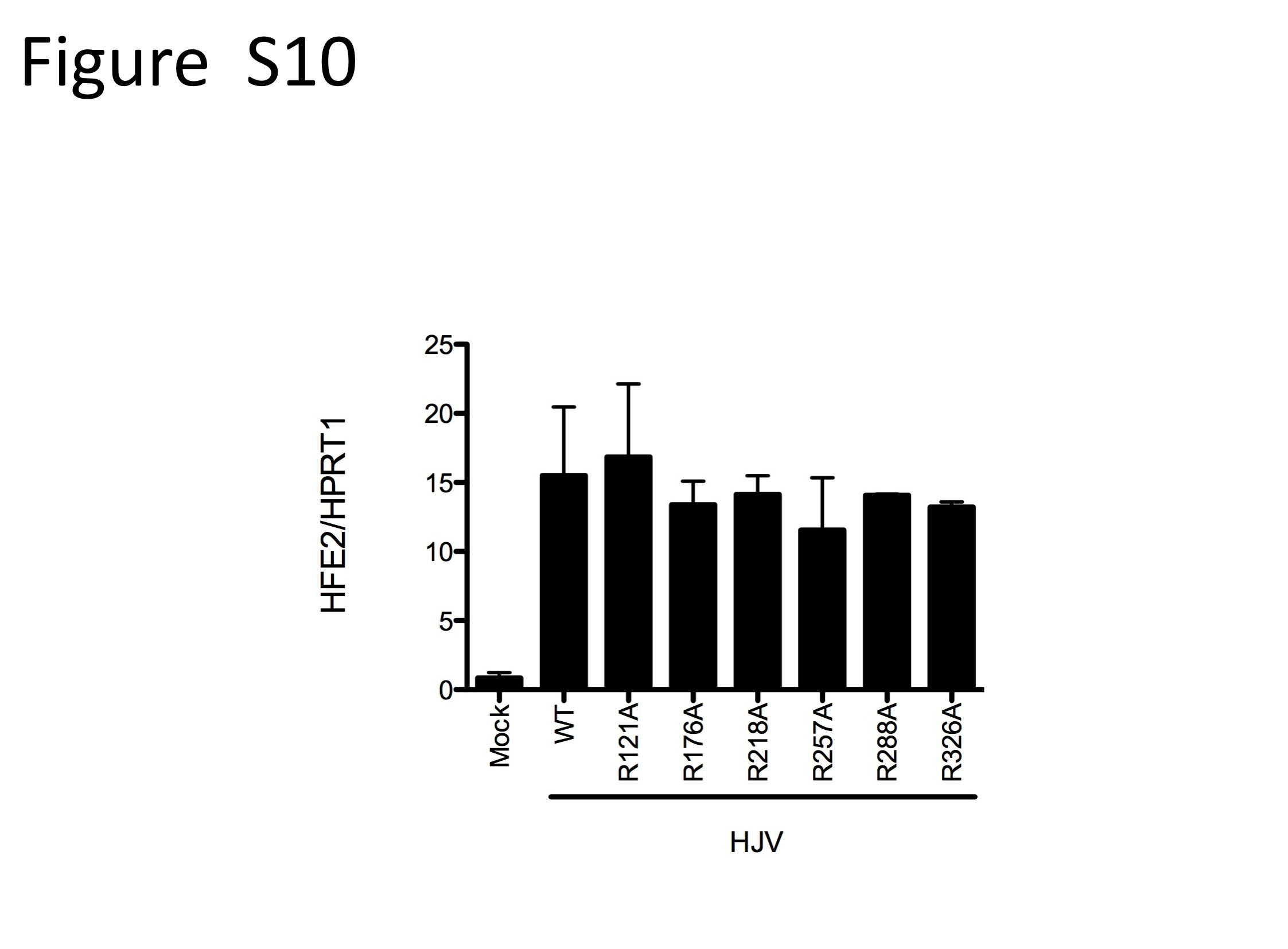

Supplement: Supplementary file 1 [file jcmm0019-0879-sd1.docx]
